# Interferon-beta expression and type I interferon receptor signaling of hepatocytes prevent hepatic necrosis and virus dissemination in Coxsackievirus B3-infected mice

**DOI:** 10.1371/journal.ppat.1007235

**Published:** 2018-08-03

**Authors:** Wolfgang Koestner, Julia Spanier, Tanja Klause, Pia-K. Tegtmeyer, Jennifer Becker, Vanessa Herder, Katharina Borst, Daniel Todt, Stefan Lienenklaus, Ingo Gerhauser, Claudia N. Detje, Robert Geffers, Martijn A. Langereis, Florian W. R. Vondran, Qinggong Yuan, Frank J. M. van Kuppeveld, Michael Ott, Peter Staeheli, Eike Steinmann, Wolfgang Baumgärtner, Frank Wacker, Ulrich Kalinke

**Affiliations:** 1 Institute for Radiology, Hannover Medical School, Hannover, Germany; 2 Institute for Experimental Infection Research, TWINCORE, Centre for Experimental and Clinical Infection Research, a joint venture between the Helmholtz Centre for Infection Research, Braunschweig, and the Hannover Medical School, Hannover, Germany; 3 Department of Pathology, University of Veterinary Medicine Hannover, Hannover, Germany; 4 Department of Molecular and Medical Virology, Ruhr University Bochum, Bochum, Germany; 5 Institute for Laboratory Animal Science and Central Animal Facility, Hannover Medical School, Hannover, Germany; 6 Helmholtz Centre for Infection Research, Genome Analytics Research Group, Braunschweig, Germany; 7 Virology Division, Department of Infectious Diseases and Immunology, Faculty of Veterinary Medicine, Utrecht University, Utrecht, The Netherlands; 8 ReMediES, Department of General, Visceral and Transplantation Surgery, Hannover Medical School, and German Centre for Infection Research, Hannover-Braunschweig, Germany; 9 Institute for Cell and Gene Therapy, TWINCORE, Centre for Experimental and Clinical Infection Research, a joint venture between the Helmholtz Centre for Infection Research, Braunschweig, and the Hannover Medical School, Hannover, Germany; 10 Institute for Virology, Medical Center University of Freiburg, Freiburg, Germany; 11 Faculty of Medicine, University of Freiburg, Freiburg, Germany; University of North Carolina at Chapel Hill School of Medicine, UNITED STATES

## Abstract

During Coxsackievirus B3 (CVB3) infection hepatitis is a potentially life threatening complication, particularly in newborns. Studies with type I interferon (IFN-I) receptor (IFNAR)-deficient mice revealed a key role of the IFN-I axis in the protection against CVB3 infection, whereas the source of IFN-I and cell types that have to be IFNAR triggered in order to promote survival are still unknown. We found that CVB3 infected IFN-β reporter mice showed effective reporter induction, especially in hepatocytes and only to a minor extent in liver-resident macrophages. Accordingly, upon *in vitro* CVB3 infection of primary hepatocytes from murine or human origin abundant IFN-β responses were induced. To identify sites of IFNAR-triggering we performed experiments with *Mx* reporter mice, which upon CVB3 infection showed massive luciferase induction in the liver. Immunohistological studies revealed that during CVB3 infection MX1 expression of hepatocytes was induced primarily by IFNAR-, and not by IFN-III receptor (IFNLR)-triggering. CVB3 infection studies with primary human hepatocytes, in which either the IFN-I or the IFN-III axis was inhibited, also indicated that primarily IFNAR-, and to a lesser extent IFNLR-triggering was needed for ISG induction. Interestingly, CVB3 infected mice with a hepatocyte-specific IFNAR ablation showed severe liver cell necrosis and ubiquitous viral dissemination that resulted in lethal disease, as similarly detected in classical IFNAR^-/-^ mice. In conclusion, we found that during CVB3 infection hepatocytes are major IFN-I producers and that the liver is also the organ that shows strong IFNAR-triggering. Importantly, hepatocytes need to be IFNAR-triggered in order to prevent virus dissemination and to assure survival. These data are compatible with the hypothesis that during CVB3 infection hepatocytes serve as important IFN-I producers and sensors not only in the murine, but also in the human system.

## Author summary

CVB3 belongs to human enteroviruses and is transmitted through the fecal-oral route. Infections with CVB3 are mostly unnoticed or cause flu-like symptoms, however, they can also cause severe disease, such as myocarditis, pancreatitis, and hepatitis. Although CVB3 does not efficiently trigger plasmacytoid dendritic cells, which are the main IFN-I producers in many other virus infections, IFNAR signaling plays a crucial role in CVB3 control. Therefore, we investigated which cells are stimulated to produce IFN-I following CVB3 infection and which cell types have to be IFNAR-triggered in order to confer anti-viral protection. We found that upon CVB3 infection IFN-β was mainly expressed within the liver, especially by hepatocytes and not by liver resident macrophages. This was corroborated by *in vitro* CVB3 infection experiments with primary murine and human hepatocytes. Interestingly, IFNAR signaling of hepatocytes was required to control the virus. Collectively, our data indicate that hepatocytes, and not immune cells, are the key innate effector cells that are relevant for the control of CVB3 infection.

## Introduction

Coxsackievirus B3 (CVB3) is a single-stranded RNA virus that belongs to the genus of human Enterovirus [1]. CVB3 infections are very common, especially in children and neonates, and mostly cause only mild disease. However, occasionally also severe disease with fatal outcome, such as myocarditis, meningoencephalitis, or hepatitis, can occur. In adults, only few cases of CVB3-induced hepatic necrosis have been reported [2, 3], whereas in neonates CVB3-induced hepatitis is more frequent [4]. Especially in Taiwan several outbreaks of CVB3 infections with predominant hepatitis and occasionally lethal outcome of up to 30% have been reported [5–7]. Recently, the analysis of murine neonates revealed that increased susceptibility to CVB3 correlated with high expression of the Coxsackievirus-adenovirus receptor in the liver, which decreased with age [8]. Moreover, lower expression of IFN-α during the first year of human life might contribute to increased infection susceptibility in infants [9].

Type I IFN receptor (IFNAR)-deficient (IFNAR^-/-^) mice succumbed to CVB3 infection within days [10]. Such mice showed early elevated serum markers of fulminant liver damage and high virus titers in the liver [10]. Similarly, also in CVB3-infected IFN-β-deficient mice elevated virus titers were detected [11], whereas in these mice liver injury was not reported and mortality was related to cardiomyocyte necrosis. However, so far it remains elusive, which cells critically depend on IFNAR triggering in order to control liver infection. Furthermore, it is not known, which cell types produce the protective IFN-I. Recently, Lind *et al*. demonstrated reduced CVB3 infection of primary human hepatocytes after treatment with IFN-III [12]. However, whether IFN-III plays an important role in the pathogenesis of CVB3 infection is still elusive.

Here, we investigated spatio-temporal conditions of IFN-β induction and IFNAR signaling during CVB3 infection, and we addressed the role of IFN-III. We found that hepatocytes, and not myeloid cells, were the main IFN-β producers that also had to be IFNAR triggered in order to restrict viral dissemination and to promote survival. Furthermore, in the murine system type III IFN receptor (IFNLR) signaling of hepatocytes was dispensable for the control of fulminant hepatitis and to resolve CVB3 infection. *In vitro* CVB3 infection experiments with primary human hepatocytes revealed a dominant role of the IFN-I axis for ISG induction.

## Results

### CVB3 infection induces abundant IFN-I and IFN-III responses in liver and pancreas

**Fig 1:**
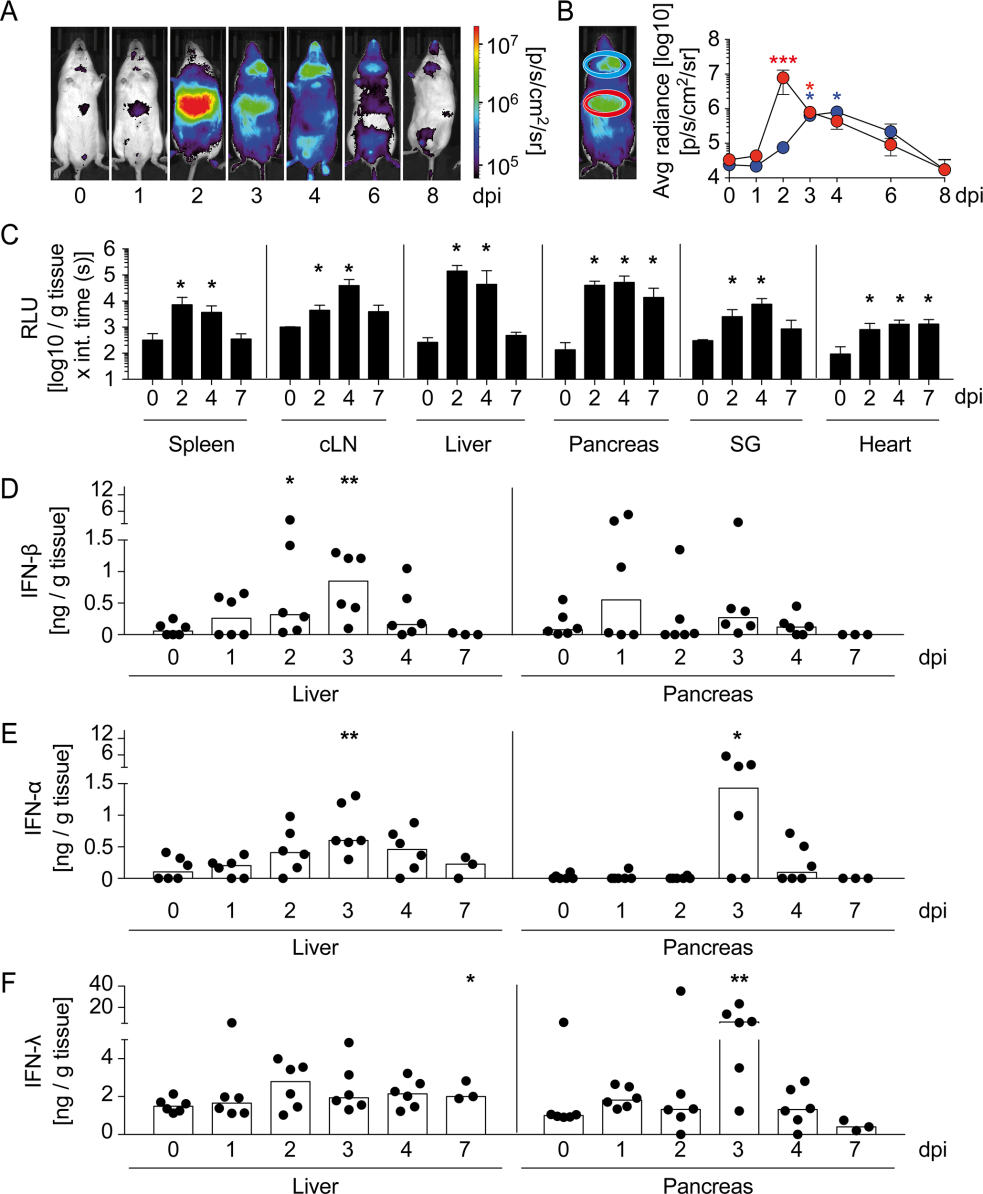
During CVB3 infection abundant IFN-I and IFN-III responses are induced in liver and pancreas. IFN-β^wt/Δβ-luc^ mice (A-C) or C57BL/6 mice (D-F) were infected i.p. with 2 × 10^4^ PFU CVB3 and (A) luciferase activity was analyzed at the indicated time points by *in vivo* imaging. One representative mouse is shown. (B) Quantification of bioluminescence imaging in selected regions of interest (ROI) for upper abdominal (red) and cervical region (blue). Results are pooled from three independent experiments (n = 3–6). Values are mean ± SD. One-Way ANOVA test was used for statistical analysis. (C) IFN-β^wt/Δβ-luc^ mice were perfused with PBS at 0, 2, 4, and 7 dpi (n = 3–9). Spleen, cervical lymph nodes (cLN), liver, pancreas, salivary glands, and heart were prepared, homogenized, and the reporter activity was determined. Values are mean ± SD. Mann-Whitney test was used for statistical analysis. (D-F) Mice were sacrificed at 0, 1, 2, 3, 4, or 7 dpi (n = 3–6). Homogenates from liver and pancreas were assessed for (D) IFN-β, (E) IFN-α, and (F) IFN-λ protein levels by ELISA methods. Pooled data from two independent experiments are shown. Bars depict median. Mann-Whitney test was used for statistical analysis, *P < 0.05; **P < 0.01; ***P < 0.*001*.

To investigate spatio-temporal conditions of IFN-β induction upon CVB3 infection, IFN-β reporter mice [13] were intraperitoneally (i.p.) injected with 2 × 10^4^ PFU CVB3 and *in vivo* imaging was performed. Already 1 day post infection (dpi) moderate luciferase induction was detected in the abdominal region, which reached a maximum 2 dpi and then declined (Fig 1A and 1B). Other significant bioluminescence imaging signals were detected in the cervical region with a maximum on 4 dpi (Fig 1A and 1B). To more specifically identify tissues showing reporter expression, infected reporter mice were perfused with PBS, organs were dissected, and luciferase expression was analyzed *ex vivo* in organ homogenates. At 2 dpi, robust bioluminescent imaging signals were detected in liver and pancreas, whereas in secondary lymphatic organs, salivary gland, and heart less abundant signals were observed (Fig 1C). To investigate the expression of IFN-I and IFN-III, organ homogenates were assessed by ELISA. Reminiscent to the *in vivo* imaging data, we found moderate amounts of IFN-I in spleen and maximal levels in cervical lymph nodes (cLN) 3 to 4 dpi (S1 Fig). In contrast, lysates of secondary lymphoid organs contained only minimal IFN-III levels (S1 Fig). In the liver, peak levels of IFN-I and IFN-III were detected 3 and 2 dpi, respectively (Fig 1D–1F). In pancreas, maximal IFN-β induction was observed 1 dpi, whereas IFN-α and IFN-III peaked on 3 dpi (Fig 1D–1F). Thus, upon CVB3 infection abundant amounts of IFN-I and IFN-III were produced primarily in liver and pancreas.

### Within the liver hepatocytes, but not myeloid cells, are the major IFN-β producers upon CVB3 infection

**Fig 2:**
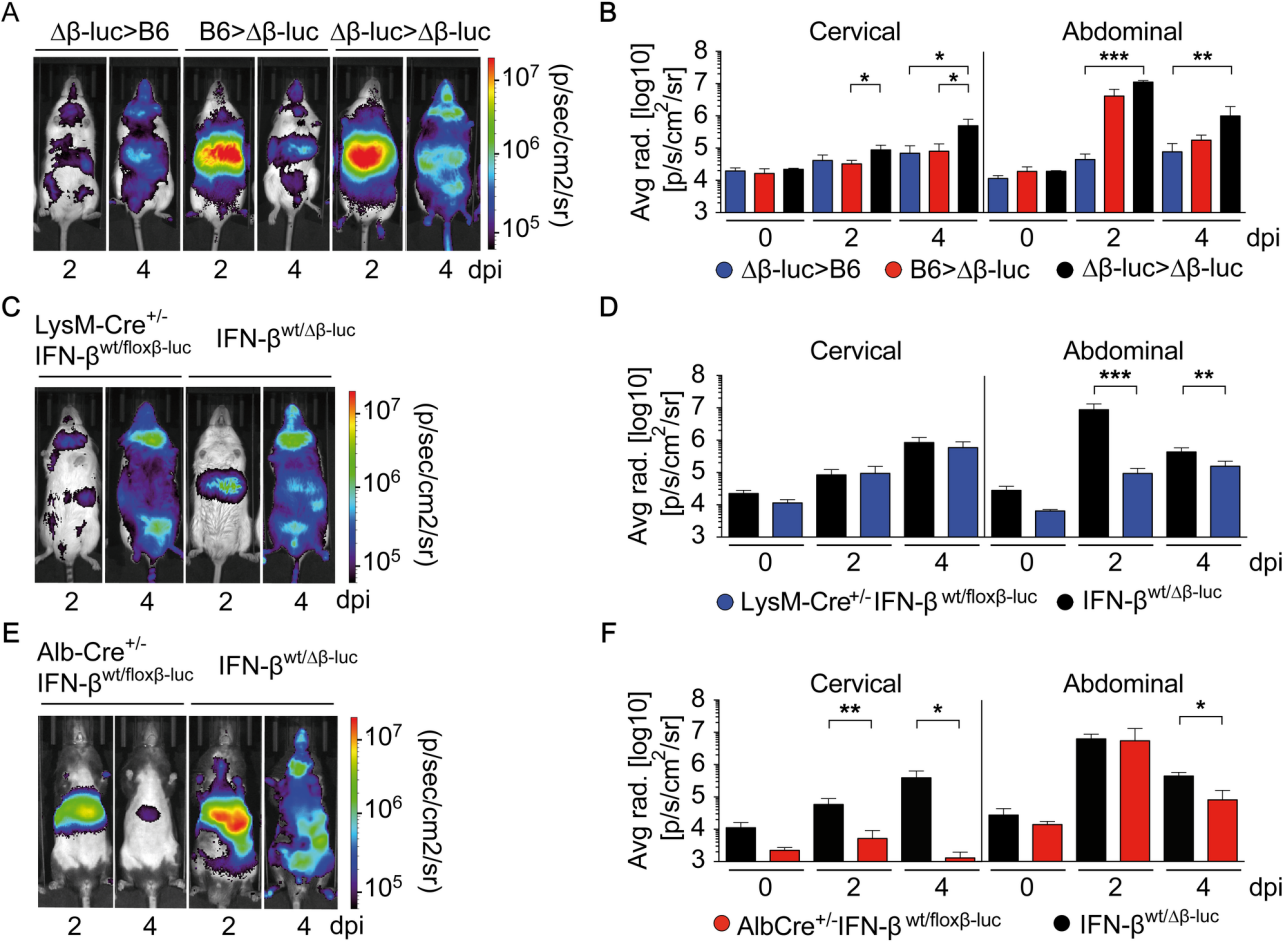
Upon CVB3 infection IFN-β induction in the liver is conferred primarily by hepatocytes. (A and B) Lethally irradiated IFN-β^wt/Δβ-luc^ (Δβ-luc) or C57BL/6 (B6) recipients were reconstituted with 1 x 10^7^ BM cells from C57BL/6 (B6>Δβ-luc, red) or IFN-β^wt/Δβ-luc^ mice (Δβ-luc>B6, blue), respectively. IFN-β^wt/Δβ-luc^ recipients reconstituted with IFN-β^wt/Δβ-luc^ BM were used as controls (Δβ-luc> Δβ-luc, black). Eight weeks after transplantation, mice were infected i.p. with 2 × 10^4^ PFU CVB3 and imaged at 0, 2, or 4 dpi (n = 3–9). (A) One representative mouse of each group is shown. (B) Quantification of *in vivo* imaging by analysis of cervical and upper abdominal regions of interest (ROI). Values are mean + SD. (C-F) Conditional IFN-β reporter mice with an activated reporter in myeloid cells (LysM-Cre^+/-^IFN-β^wt/floxβ-luc^) or in hepatocytes (Alb-Cre^+/-^IFN-β^wt/floxβ-luc^) were infected as in (A) and imaged at the indicated dpi. IFN-β^wt/Δβ-luc^ control mice with C57BL/6 albino or C57BL/6 background were used as controls, respectively. (C, E) One representative mouse of each genotype is shown. (D, F) Quantification of *in vivo* imaging (n = 3–9). Values are mean + SD. One-Way ANOVA test was used for statistical analysis, *P < 0.05; **P < 0.01; ***P < 0.*001*.

To address whether in the liver primarily hematopoietic cells or non-hematopoietic cells were triggered to mount IFN-β responses upon CVB3 infection, lethally irradiated C57BL/6 recipient mice were reconstituted with bone marrow (BM) from IFN-β^wt/Δβ-luc^ mice (IFN-β^wt/Δβ-luc^>C57BL/6), which resulted in BM chimeric mice that carried the IFN-β reporter only in hematopoietic cells. *In vivo* imaging of such mice after CVB3 infection revealed moderate bioluminescence imaging (BLI) signals in the abdomen 2 dpi and in the cervical region 4 dpi (Fig 2A and 2B). To investigate the contribution of non-hematopoietic cells, C57BL/6>IFN-β^wt/Δβ-luc^ BM chimeric mice were studied. In such mice abundant BLI signals were observed in the liver that were similar to the ones detected in IFN-β^wt/Δβ-luc^>IFN-β^wt/Δβ-luc^ control mice (Fig 2A and 2B). Thus, upon CVB3 infection IFN-β expression within the liver was contributed primarily by non-hematopoietic cells. To further elucidate the identity of IFN-β expressing cells in the liver, myeloid- and hepatocyte-specific IFN-β reporter mice were analyzed (LysM-Cre^+/-^IFN-β^wt/floxβ-luc^ and Alb-Cre^+/-^IFN-β^wt/floxβ-luc^ mice, respectively). CVB3-infected LysM-Cre^+/-^IFN-β^wt/floxβ-luc^ mice showed moderate reporter induction in the abdominal region, whereas the induction in the cervical region was comparable with that detected in ubiquitous IFN-β^wt/floxβ-luc^ reporter mice (Fig 2C and 2D). These data indicated that in the cervical region, but not in the liver, IFN-β induction was conferred by myeloid cells. Upon CVB3 infection of Alb-Cre^+/-^IFN-β^wt/floxβ-luc^ mice similar BLI signals were detected in the abdominal region as in IFN-β^wt/Δβ-luc^ mice (Fig 2E and 2F). Thus, within the liver primarily hepatocytes conferred IFN-β responses upon CVB3 infection.

### Upon CVB3 infection IFNAR, but not IFNLR, signaling mediates Mx gene induction in hepatocytes

**Fig 3:**
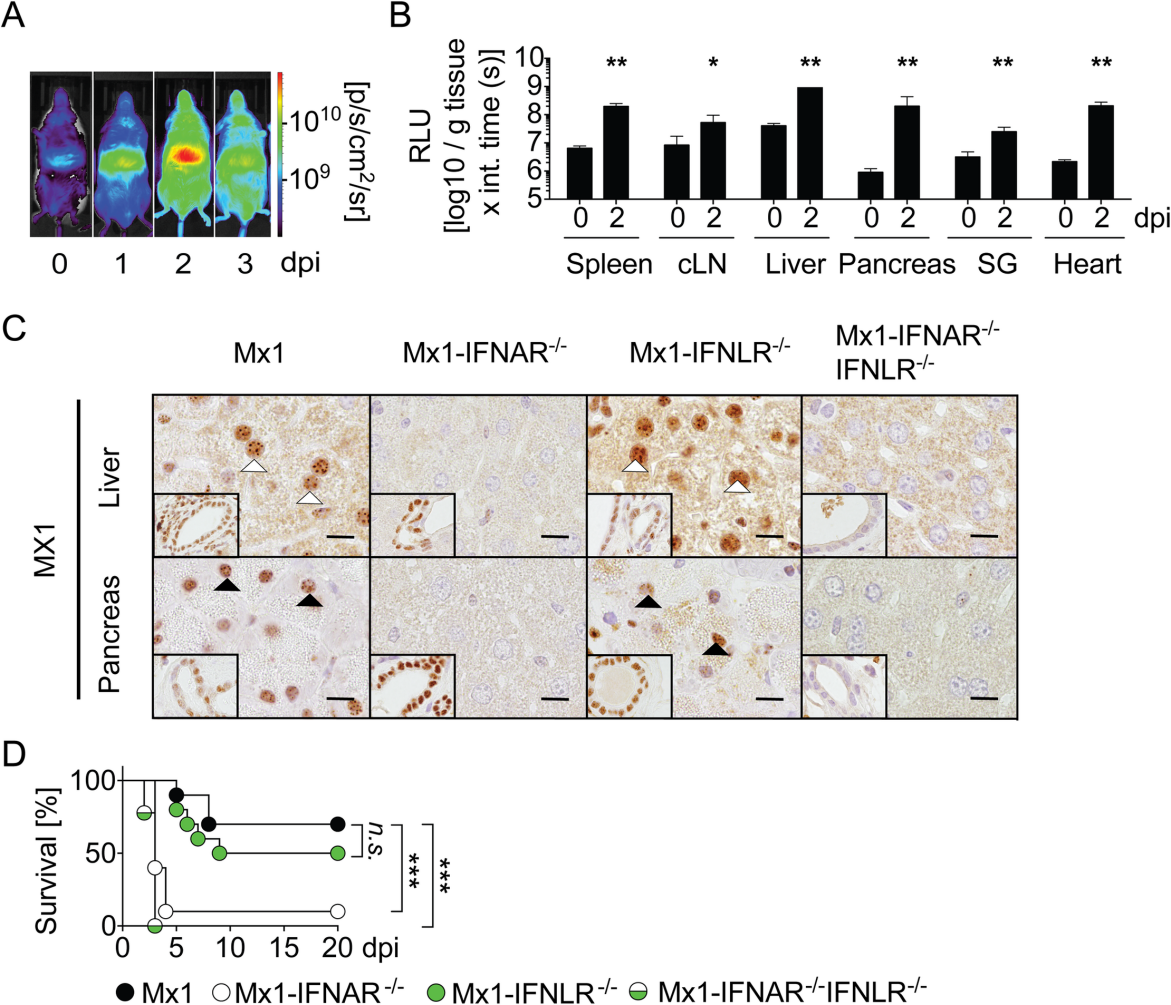
Upon CVB3 infection IFNAR but not IFNLR signaling mediates Mx induction in hepatocytes. (A and B) Mx2-Luc reporter mice were i.p. infected with 2 × 10^4^ PFU CVB3 and *in vivo* imaging was performed (A) at the indicated dpi (n = 3; one representative mouse is shown). (B) 0 and 2 dpi Mx2-Luc reporter mice were perfused with PBS, organs were dissected, homogenates of spleen, cervical lymph nodes (cLN), liver, pancreas, salivary gland, and heart were assessed for reporter activity *in vitro* (n = 6). Values are mean + SD. Mann-Whitney test was used for statistical analysis. (C) Mx1, Mx1-IFNAR^-/-^, Mx1-IFNLR^-/-^, and Mx1-IFNAR^-/-^IFNLR^-/-^ mice were infected i.p. with 2 × 10^4^ PFU CVB3 and sacrificed 2 dpi. Immunohistochemical analysis of MX1 expression within sections of liver (line 1) and pancreas (line 2). MX1 positive nuclei of hepatocytes (line 1) or acinar cells (line 2) are highlighted by white and black arrow heads, respectively. Inserts show MX1 immunoreactivity of nuclei of bile ducts (line 1) or pancreatic ducts (line 2). Bars = 10 μm. Representative sections of one mouse out of four are shown. (D) Mx1, Mx1-IFNAR^-/-^, Mx1-IFNLR^-/-^, and Mx1-IFNAR^-/-^IFNLR^-/-^ mice were infected as in (C) and monitored for survival (n = 9–10). *P < 0.*05*, **P < 0.*01*, ***P < 0.*001*, *n*.*s*. *= not statistically significant*.

To assess sites at which IFN responses exhibit biological function, Mx2Luc-reporter mice carrying a firefly luciferase gene under the control of the IFN-I and IFN-III-responsive *Mx2* promoter [14] were evaluated upon CVB3 infection. In line with previous reports [14], constitutive expression of the reporter was observed in non-infected mice in the liver (Fig 3A). Upon CVB3 infection, significant reporter gene induction was detected in the upper abdomen (Fig 3A). Notably, in CVB3 infected Mx2Luc mice the kinetics of the reporter induction in the upper abdominal region was similar to that detected in IFN-β reporter mice (compare Fig 1A and Fig 3A). *Ex vivo* analysis revealed *Mx2*-dependent reporter induction in all organs analyzed with the highest values detected in the liver (Fig 3B). To investigate *Mx* induction on the cellular level, Mx1 mice carrying a functional *Mx1* gene were used [15]. In contrast to uninfected controls (S2A Fig), CVB3-infected Mx1 mice showed MX1 protein induction in hepatocytes and bile duct cells 2 dpi (Fig 3C). Interestingly, no MX1 expression was detected in hepatocytes of CVB3-infected Mx1-IFNAR^-/-^ mice (Fig 3C) indicating that *in vivo* IFNLR triggering of hepatocytes did not play a major role. In contrast, bile duct epithelial cells showed MX1 expression in CVB3-infected Mx1-IFNAR^-/-^ mice, which indicated triggering by locally produced IFN-III. MX1 expression in hepatocytes and bile duct epithelial cells in infected Mx1-IFNLR^-/-^ mice was similar to that detected in infected Mx1 mice (Fig 3C) showing that both hepatocytes and bile duct epithelial cells responded to IFN-I. As expected, neither hepatocytes nor bile duct epithelial cells showed MX1 protein induction in CVB3-infected Mx1-IFNAR^-/-^IFNLR^-/-^ mice (Fig 3C). Pancreatic acinar cells showed MX1 expression in infected Mx1-IFNLR^-/-^, but not in Mx1-IFNAR^-/-^ mice (Fig 3C). Thus, acinar cells responded to IFN-I, but not to IFN-III. In contrast, pancreatic duct epithelial cells showed MX1 protein induction in both Mx1-IFNLR^-/-^ and Mx1-IFNAR^-/-^ mice indicating that these cells responded to IFN-I and IFN-III (Fig 3C). Moreover, liver and pancreas of CVB3 infected as well as uninfected Mx1-WT, Mx1-IFNAR^-/-^, Mx1-IFNLR^-/-^ and Mx1-IFNAR^-/-^IFNLR^-/-^ mice were analyzed for ISG15 expression by immunohistology (S2B and S2C Fig). These data revealed an overall very similar picture as the analysis of MX1 expression. Since Mx1-IFNLR^-/-^ mice were as resistant to CVB3 infection as Mx1 controls, whereas Mx1-IFNAR^-/-^ and Mx1-IFNAR^-/-^IFNLR^-/-^ mice succumbed to CVB3 infection (Fig 3D), IFN-III did not seem to play a major role in the pathogenesis of murine CVB3 infection. Furthermore, histological analysis of the liver of infected Mx1-IFNLR^-/-^ mice did not reveal inflammation and necrosis (S3A and S3B Fig). Taken together, CVB3 induces non-redundant IFNAR signaling in hepatocytes and acinar cells, whereas in bile duct epithelial cells and pancreatic duct epithelial cells concomitant IFNAR and IFNLR signaling was detected.

### IFNAR signaling in hepatocytes prevents CVB3-induced hepatitis and virus dissemination

**Fig 4:**
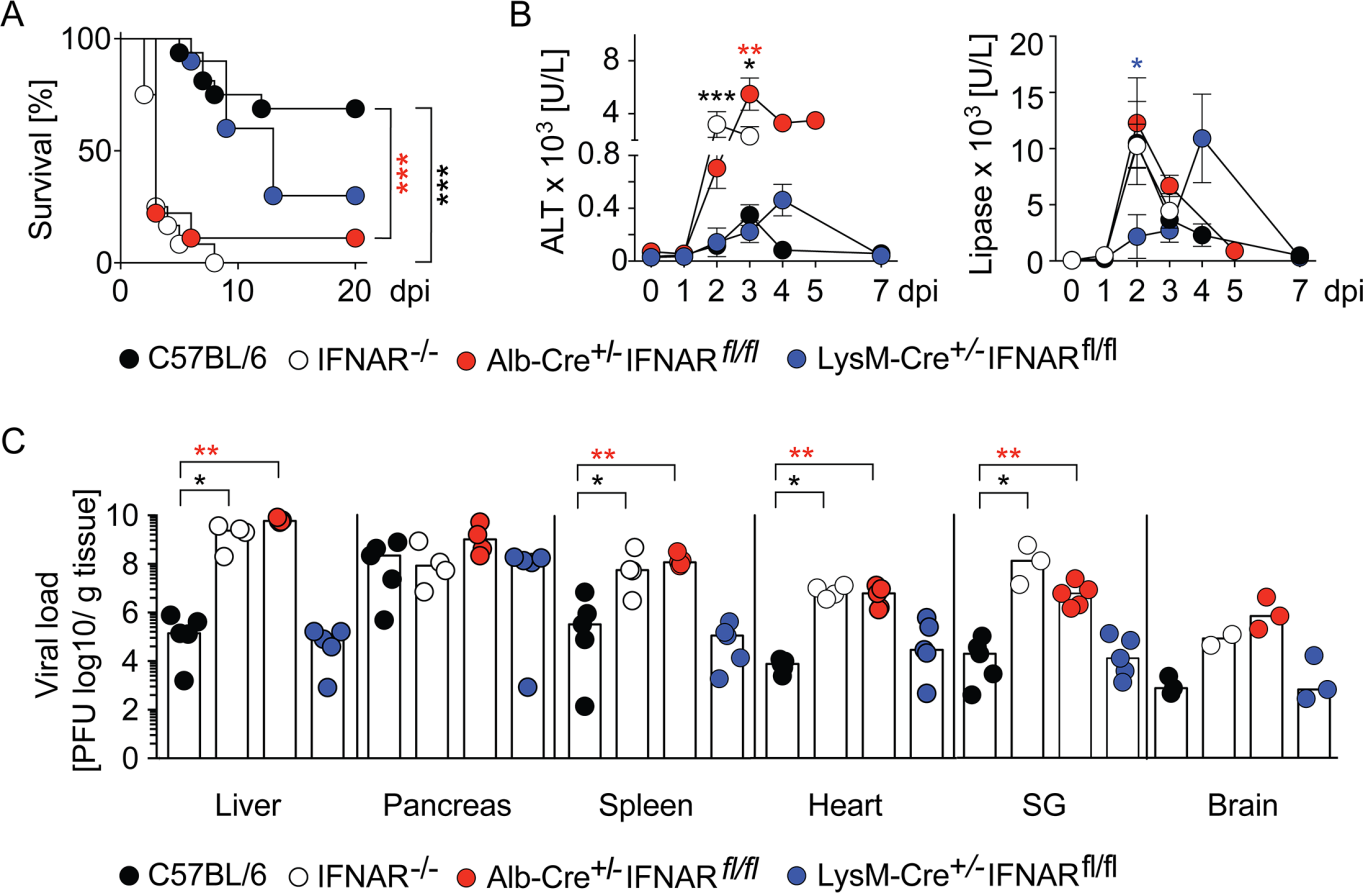
Mice with a hepatocyte-specific IFNAR deletion succumb to CVB3 infection and show ubiquitous virus dissemination. C57BL/6 (black), IFNAR^-/-^ (white), Alb-Cre^+/-^IFNAR^fl/fl^ (red), and LysM-Cre^+/-^IFNAR^fl/fl^ (blue) mice were infected i.p with 2 × 10^4^ PFU CVB3 and (A) monitored for survival. Mice that showed severe clinical symptoms or more than 20% body weight lost were sacrificed (n = 9–16). Differences between the groups were determined using log-rank statistics. (B) Alanine aminotransferase (ALT) or lipase levels were determined from serum at the indicated dpi. Of note, for Alb-Cre^+/-^IFNAR^fl/fl^ mice values at 4 and 5 dpi from only one surviving mouse per day are depicted. Values are mean ± SEM (n = 3–13). For statistical analysis, IFNAR^-/-^, Alb-Cre^+/-^IFNAR^fl/fl^, or LysM-Cre^+/-^IFNAR^fl/fl^ mice were compared with C57BL/6 mice by One-Way ANOVA test. (C) Graphs depict virus titers in liver, pancreas, spleen, heart, salivary gland (SG), and brain based on plaque formation 3 dpi (n = 2–5). Bars depict mean. Mann-Whitney test was used for statistical analysis, *P < 0.05; **P < 0.*01*. ***P < 0.*001*.

As similarly published before [10], IFNAR^-/-^ mice succumbed to CVB3 infection within days, whereas C57BL/6 control mice did not show overt signs of disease (Fig 4A). Nevertheless, several WT control mice had to be sacrificed due to body weight loss of more than 20% (Fig 4A). To investigate the impact of IFNAR signaling of myeloid cells within the liver, we generated LysM-Cre^+/-^IFNAR^fl/fl^ mice with a selective IFNAR deletion in myeloid cells. Upon CVB3 infection, most of these mice appeared overall healthy during the observation period, whereas some mice had to be sacrificed due to body weight loss of more than 20% (Fig 4A). Thus, IFNAR signaling in myeloid cells did not play a dominant role in the protection against CVB3 infection. Likewise, also Alb-Cre^+/-^IFNAR^fl/fl^ mice with a hepatocyte-specific IFNAR-deletion were CVB3-infected and interestingly these mice showed a similarly enhanced sensitivity to CVB3 infection as detected in IFNAR^-/-^ mice (Fig 4A). In infected Alb-Cre^+/-^IFNAR^fl/fl^ and IFNAR^-/-^ mice dramatically enhanced ALT levels were found (Fig 4B), whereas infected WT controls and LysM-Cre^+/-^IFNAR^fl/fl^ mice showed moderately increased ALT levels (Fig 4B). Thus, CVB3-induced hepatitis correlated with impaired IFNAR signaling of hepatocytes. In contrast, all mice of the different genotypes analyzed showed a similar extent of pancreatitis, as indicated by enhanced lipase levels, although the kinetics of lipase level increase was delayed in LysM-Cre^+/-^IFNAR^fl/fl^ mice (Fig 4B). In conclusion, in WT mice the development of pancreatitis and massive pancreatic viral replication (Fig 4C) were independent of functional IFNAR signaling.

Next we assessed the impact of IFNAR signaling of hepatocytes and myeloid cells on viral dissemination. In the liver of infected WT mice viral titers increased by approximately 6 log until 3 dpi, whereas in IFNAR^-/-^ mice they increased by approximately 10 log (Fig 4C). Notably, also Alb-Cre^+/-^IFNAR^fl/fl^ mice showed massively increased viral titers in liver, spleen, heart, salivary gland, and brain (Fig 4C). In contrast, viral titers in organs of infected LysM-Cre^+/-^IFNAR^fl/fl^ mice were similar as in organs of C57BL/6 mice (Fig 4C). Thus, IFNAR signaling of hepatocytes played a key role in restricting viral dissemination.

### Mice with a hepatocyte-specific IFNAR ablation show infection of hepatocytes and severe liver cell necrosis

**Fig 5:**
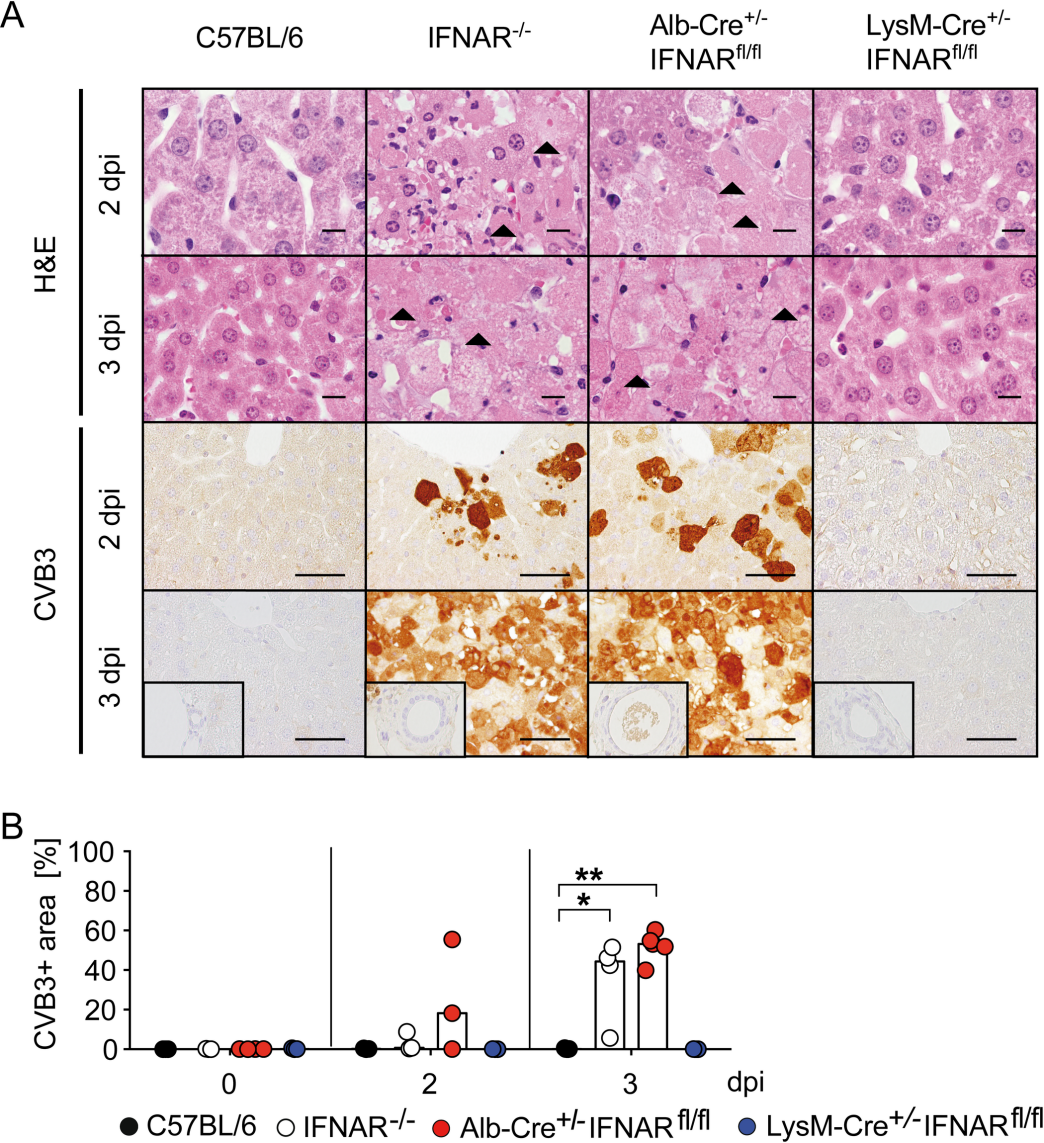
Mice with a hepatocyte-specific IFNAR ablation show infection of hepatocytes and severe hepatocellular necrosis. C57BL/6, IFNAR^-/-^, Alb-Cre^+/-^IFNAR^fl/fl^, and LysM-Cre^+/-^IFNAR^fl/fl^ mice were infected i.p. with 2 × 10^4^ PFU CVB3 and sacrificed 0, 2, or 3 dpi (n = 3–5). (A) Liver sections were H&E stained (line 1 and 2; bars = 10 μm) or subjected to CVB3-specific immunohistochemistry (line 3 and 4; bars = 50 μm). Arrowheads highlight necrotic hepatocytes (coagulative necrosis). Inserts show a lack of immunoreactivity in biliary ducts. (B) Quantification of area of infected liver tissue in CVB3-immunohistochemistry sections was performed by AnalySIS 3.2 software (n = 3–5). Bars depict median. Mann-Whitney test was used for statistical analysis, *P < 0.05; **P < 0.*01*.

Next, we assessed the impact of myeloid cell or hepatic IFNAR ablation on liver pathology upon CVB3 infection. In histological analysis no signs of liver damage were detected in CVB3-infected C57BL/6 mice, indicating that the moderately elevated ALT levels did not translate into histopathological liver damage (Fig 5A and 5B). Similarly, also liver sections from infected LysM-Cre^+/-^IFNAR^fl/fl^ mice did not show pathological changes (Fig 5A and 5B). In contrast, IFNAR^-/-^ mice developed focal liver cell necrosis 2 dpi (Fig 5A, line 1) and widespread and severe coagulative hepatic necrosis at 3 dpi (Fig 5A, line 2). Notably, also Alb-Cre^+/-^IFNAR^fl/fl^ mice developed liver cell necrosis (Fig 5A, line 1). Interestingly, necrosis in CVB3-infected IFNAR^-/-^ and Alb-Cre^+/-^IFNAR^fl/fl^ mice was not accompanied by infiltration of immune cells (Fig 5A, line 1 and 2).

Immunohistochemical analysis of the liver from CVB3-infected IFNAR^-/-^ and Alb-Cre^+/-^IFNAR^fl/fl^ mice revealed preferential infection of hepatocytes, whereas hepatocyte infection was absent in C57BL/6 and LysM-Cre^+/-^IFNAR^fl/fl^ mice (Fig 5A). The area of CVB3-infected liver tissue correlated with the severity of liver injury and was similar in Alb-Cre^+/-^IFNAR^fl/fl^ and IFNAR^-/-^ mice (Fig 5B). Interestingly, bile duct epithelial cells did not show any abnormalities in light microscopy and were not infected, even in mouse strains in which this cell subset was IFNAR-deficient (Fig 5A, inserts). In accordance with the elevated lipase levels, all CVB3-infected genotypes displayed infection of pancreatic acinar cells and developed a widespread necrosis of the exocrine pancreas, while pancreatic islet and duct epithelial cells were not affected (S4 Fig). Taken together, IFNAR signaling of hepatocytes was necessary to prevent hepatocyte infection and necrosis, whereas necrosis of the exocrine pancreas was not affected by IFNAR signaling.

### CVB3-infected primary murine and human hepatocytes mount IFN-β responses that confer ISG induction

**Fig 6:**
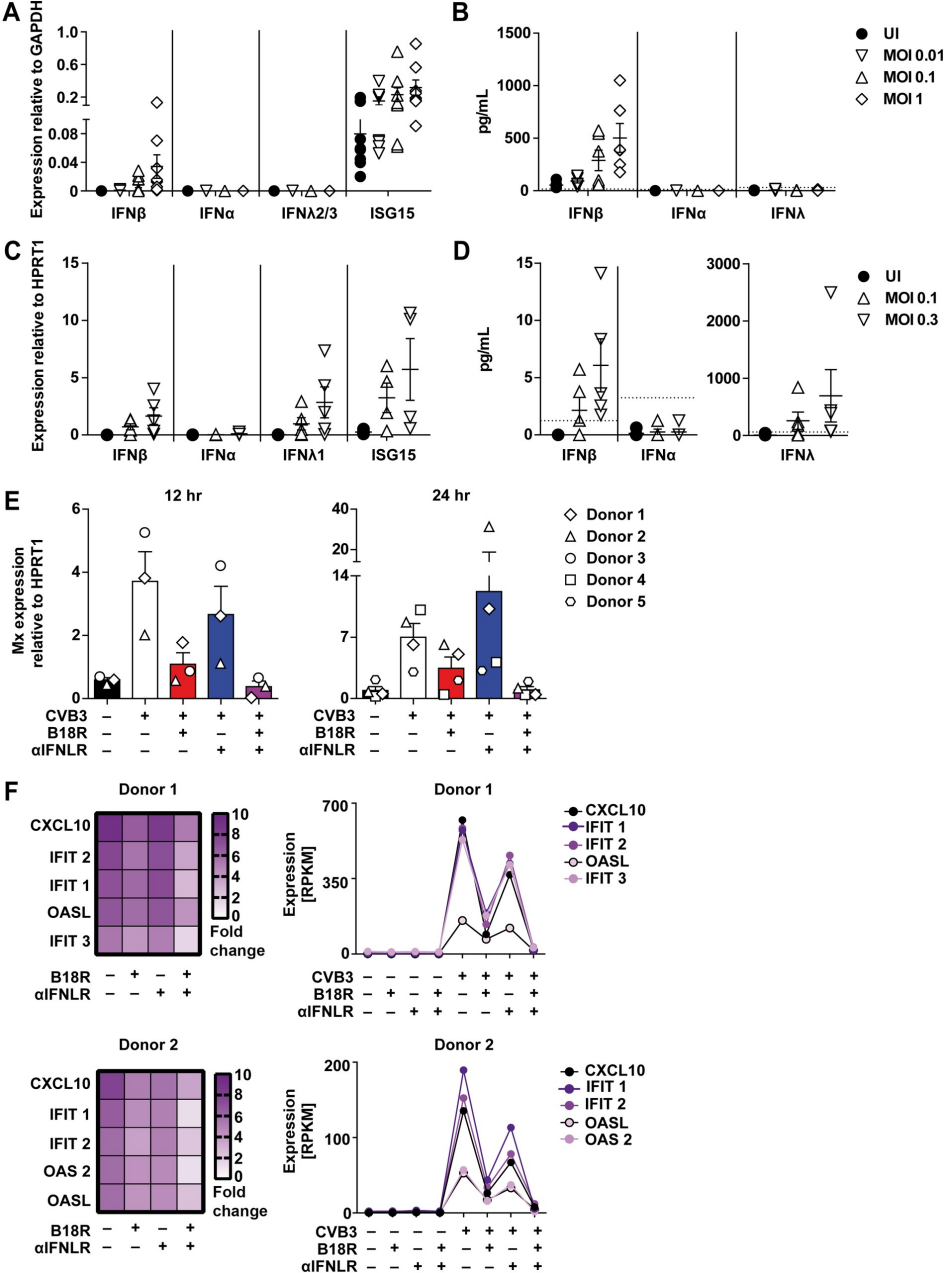
*In vitro* CVB3 infected murine and human primary hepatocytes mount IFN-β responses that are essential for robust ISG induction. (A and B) *In vitro* cultured primary hepatocytes from C57BL/6 mice were CVB3 infected in triplicates at MOI 0.01, 0.1, or 1 and after 24 hr incubation supernatants and cells were harvested. (A) *Ifn-β*, *Ifn-α*, *Ifn-λ2/3* as well as *Isg15* mRNA expression levels were analyzed by qPCR and (B) IFN-β, IFN-α, and IFN-λ3 levels were determined from supernatants by ELISA methods. Pooled data from 4 similar experiments are shown (n ≥ 1). Bars depict mean + SEM. Dotted lines indicate the detection limits of 15.6 pg/mL IFN-β, 12.5 pg/mL IFN-α, and 30.625 pg/mL IFN-λ3. (C and D) Human primary hepatocytes were infected with CVB3 at MOI 0.1 or 0.3 and after 24 hr incubation supernatants and cells were harvested. (C) mRNA levels of *Ifn-β*, *Ifn-α*, *Ifn-λ1* as well as *Isg15* were analyzed by qPCR. (D) IFN-β, IFN-α, and IFN-λ1/2/3 levels were determined by ELISA methods. Pooled data from five similar experiments are shown. Bars depict mean + SEM. Dotted lines indicate the detection limits of 1.2 pg/mL IFN-β, 3.2 pg/mL IFN-α, and 62.5 pg/mL IFN-λ1/2/3. Primary human hepatocytes from donor 1, 2, 3, 4, and 5 were CVB3 infected at MOI 0.1 for 2 hr, washed with PBS, treated either with B18R, αFNLR, or both and incubated for 12 hr for donor 1–3 and 24 hr for donor 1–5. Cells were harvested and (E) Mx mRNA levels were analyzed by qPCR. (F) Total RNA of donor 1 and 2 after 24 hpi was additionally analyzed by RNAseq and the expression of 268 ISGs was studied. The induction of the 5 most abundantly upregulated ISGs are shown as [fold change] relative to uninfected controls in a heat map (left) and as a graph showing total expression levels in [Reads Per Kilobase of transcript per Million mapped reads, RPKM] (right).

To further address whether the IFN-β detected in liver homogenates originated from hepatocytes, as implied by the *in vivo* imaging results (Fig 2), primary murine hepatocytes were infected with CVB3 and analyzed for IFN-I and IFN–III mRNA and protein expression. Of note, the preparation protocol yielded hepatocytes with a purity of approximately 90%, as determined by FACS analysis (S5A and S5B Fig). Upon CVB3 infection, primary murine hepatocytes indeed showed *Ifn-β* mRNA upregulation, whereas upregulation of *Ifn-α* or *Ifn-λ2/3* mRNA was not detected (Fig 6A). Moreover, also the expression of *Isg15* was upregulated, indicating autocrine IFNAR triggering of the hepatocytes (Fig 6A). In accordance with these results, IFN-β but not IFN-α or IFN-λ protein levels were detected in supernatants (Fig 6B). To study conditions in the human system, primary human hepatocytes were CVB3 infected. Indeed, the virus replicated in these cells, as indicated by increased virus titers detected in the supernatant after 12, 24, and 48 hpi (S5D Fig). Similar to primary murine hepatocytes, qPCR analysis of primary human hepatocytes 24 hr after CVB3 infection showed upregulation of *Ifn-β* and of *Isg15* mRNA expression, but not of *Ifn-α* mRNA expression (Fig 6C). In contrast to murine hepatocytes, human hepatocytes additionally showed upregulated *Ifn-λ1* mRNA expression (Fig 6C). This observation was in accordance with a previous study that showed IFN-λ production of CVB3 infected primary human hepatocytes that conferred ISG induction and mediated antiviral protection [12]. We detected only very low amounts of IFN-β protein in the supernatant of CVB3 infected primary human hepatocytes, while IFN-III levels were very abundant (Fig 6D and S5F Fig).

To further address the relative functional relevance of the IFN-I vs. the IFN-III axis during CVB3 infection of primary human hepatocytes, we inhibited the IFN-I and/or the IFN-III axis by treatment with either recombinant B18R, the soluble IFN-I receptor of vaccinia virus Ankara, and/or recombinant anti-interferon lambda receptor 1 antibody (αIFNLR), respectively. In control experiments human hepatocytes were stimulated with either 1 ng/mL recombinant IFN-β or 100 U/mL IFN-α, or 10 ng/mL IFN-λ1, 10 ng/mL IFN-λ2, or 30 ng/mL IFN-λ3 and treated with recombinant B18R and/or recombinant anti-interferon lambda receptor 1 antibody (αIFNLR). As expected, B18R and αIFNLR treatment efficiently inhibited IFN-I and IFN-III mediated Mx and ISG15 induction in hepatocytes, whereas B18R and/or αIFNLR treatment alone did not have any effect (S5C Fig). Next we CVB3 infected human hepatocytes for 2 hr, washed with PBS, treated with B18R and/or αIFNLR and then analyzed the RNA induction by qPCR. These experiments revealed that infected human hepatocytes showed abundant Mx induction at 12, 24, and 48 hpi, whereas B18R treatment reduced this induction, while αIFNLR had only a minor effect (Fig 6E and S5E Fig). To further study the relative effects of IFN-I and IFN-III on human hepatocytes upon CVB3 infection, RNAseq was performed on total RNA samples of two donors 24 hpi. The 5 most abundantly induced ISGs are shown (Fig 6E). As observed before, B18R treatment resulted in the reduction of ISG induction, while αIFNLR treatment had only a modeate effect. Upon combined treatment with B18R and αIFNLR the ISG induction was almost comparable with uninfected controls (Fig 6F). These data further underscored the strong antiviral effect of IFN-I on CVB3 infected primary hepatocytes, both in the murine as well as the human system.

## Discussion

Several studies demonstrated a predominant role of IFN-I and IFNAR signaling in the pathogenesis of CVB3-induced hepatitis [16]. However, the cellular subsets that contribute to these effects are not known. Therefore, in the current study we aimed at identifying cell subsets that mount protective IFN-I responses and show IFNAR triggering upon CVB3 infection. In CVB3-infected mice, we found abundant IFN-β, IFN-α, and IFN-λ responses in the liver, of which IFN-β was produced primarily by hepatocytes. Moreover, hepatocytes showed exclusively IFNAR- and not IFNLR-dependent ISG induction in mice. Interestingly, IFNAR triggering of hepatocytes prevented severe liver cell necrosis and dissemination of the virus to other organs. CVB3 infection of human hepatocytes induced IFN-β as well as IFN-λ responses, whereas ISG expression was mainly triggered by IFN-β. These results indicate that during CVB3 infection similar mechanisms apply in the murine and the human system.

Consistent with previous reports that following CVB3 infection of mice *Ifn-β* mRNA expression was detected in the liver [17], we found early and strong induction of IFN-β protein in liver lysates. Because within the liver hepatocytes as well as myeloid cells, such as Kupffer cells or infiltrating pro-inflammatory monocytes, have been reported to be able to mount IFN-β responses [18, 19], we addressed which of the two subsets accounted for the IFN-β responses upon CVB3 infection. Our studies with BM chimeric mice, in which either hematopoietic or non-hematopoietic cells reported on IFN-β expression, as well as experiments with hepatocyte- and myeloid cell-specific conditional IFN-β reporter mice, indicated that upon CVB3 infection IFN-β induction in the liver was contributed mainly by hepatocytes, and to a minor extent by myeloid cells. In line with that, *ex vivo* isolated primary murine hepatocytes mounted substantial IFN-β responses. Thus, our data indicate that in CVB3 infection hepatocytes are important IFN-β producers in the liver. In a recent study, CVB3-infected mice with a cardiomyocyte-specific IFNAR ablation showed enhanced virus replication in the heart [20]. However, in that study it was not addressed whether locally or extra-cardiacally produced IFN-I conferred protective effects in cardiomyocytes. Here, we detected only very minor IFN-β induction in the heart, whereas early and strong IFN-I responses were found in the liver, suggesting that hepatic IFN-I responses determined whether peripheral organs, such as the heart, were assaulted or not.

In addition to IFN-β, we found early and significant IFN-α levels in liver lysates that were particularly abundant on 2 dpi, when IFN-α was also detected at low level in the serum (S1D Fig). Since plasmacytoid dendritic cells (pDC) have been reported to be main IFN-α producers in the liver [21], it is possible that hepatic IFN-α was derived from local pDC. However, upon *in vitro* incubation with CVB3, human and murine pDC were unable to mount strong IFN-α responses in the absence of opsonizing CVB3 antibodies [22]. Since it is unlikely that CVB3-specific antibodies are present as early as 2 dpi, this raises the question whether pDC could be activated *in vivo* by other mechanisms. Indeed, for other viruses it was shown that direct virus infection of pDC was not required to trigger IFN-α responses [23]. IFN-α induction was even stronger when pDC were stimulated by infected cells [24]. Thus, CVB3-infected hepatocytes might be able to trigger IFN-α production by pDC, as previously shown for HCV-infected cells [25]. Finally, also extrahepatically produced IFN-α can reach the liver via the circulation.

Livers of untreated mice showed constitutive *Mx2* induction that was further enhanced after CVB3 infection. Mainly hepatocytes and bile duct epithelial cells, but not immune cells, showed MX1 and ISG15 protein induction. MX1 and ISG15 upregulation in hepatocytes was IFNAR-, and not IFNLR-dependent, whereas bile duct epithelial cells showed IFNAR- and IFNLR-dependent MX1 and ISG15 upregulation. The moderately enhanced ISG15 induction in CVB3 infected Mx1-IFNAR^-/-^IFNLR^-/-^ mice can be explained by direct ISG15 induction through pattern recognition receptor engagement by virus associated molecular patterns, as similarly detected for other pathogens [26]. Our data highlighting the relevance of IFNAR signaling on hepatocytes during CVB3 infection are in accordance with a previous report in which upon infection with a hepatotropic influenza A virus strain MX1 induction in hepatocytes was also independent of IFNLR triggering [27].

Similar to previous studies, we also observed virus dissemination and severe liver pathology in CVB3-infected IFNAR^-/-^ mice [10]. Liver pathology was characterized by severe necrosis and infection of hepatocytes. IFNAR-triggering of myeloid cells was shown to be crucial for the control of many different virus infections [28–31]. Nevertheless, mice with a selective IFNAR ablation on myeloid cells showed normal survival of CVB3 infection and only moderate or no hepatitis, as similarly detected in WT mice. In contrast, mice with a hepatocyte-specific IFNAR-deletion showed increased virus titers in the liver and the virus disseminated to other fully IFNAR competent peripheral organs, as similarly observed in IFNAR^-/-^ mice that show an ubiquitous IFNAR deletion. Furthermore, it was shown that mice with a cardiomyocyte-specific IFNAR ablation had only increased viral titers in the heart, but not in other peripheral organs tested [20]. These data are compatible with the model that IFNAR signaling of hepatocytes is essential to control CVB3 infection and to prevent its dissemination. Therefore, it is possible that the responsiveness of hepatocytes to CVB3 infection as well as the IFN-I responsiveness of hepatocytes affect the individual risk to develop myocarditis.

These data concur with the earlier observation that CVB3-infected IFN-β^-/-^ mice showed higher virus titers in the liver than WT mice [11]. However, in CVB3-infected IFN-β^-/-^ mice no hepatitis was detected, suggesting that under such conditions IFN-α responses modulated hepatitis and thus compensated the IFN-β deficiency. Interestingly, in hepatic necrosis of CVB3-infected IFNAR^-/-^ and Alb-Cre^+/-^IFNAR^fl/fl^ mice we observed only very few infiltrating immune cells. This absence of infiltrating immune cells in hepatic necrosis was also detected in fatal cases of CVB3-infected neonates [3, 4]. A similar phenotype was also described for CVB3-infected mice with an ablation either of the cytoplasmic dsRNA sensor melanoma differentiation-associated protein 5 (MDA5) or its adaptor MDA-5-transducing mitochondrial antiviral signaling protein (MAVS) [16, 17]. It was speculated that in MAVS^-/-^ mice the induction of reduced IFN-I levels was responsible for the development of hepatocyte necrosis [16], which suggested the need of MAVS-dependent signaling in hepatocytes for the induction of IFN-I responses.

Interestingly, upon CVB3 infection WT, IFNAR^-/-^, Alb-CreIFNAR^fl/fl^, and LysM-CreIFNAR^fl/fl^ mice showed high viral loads in the pancreas and all the different mouse strains tested displayed similar signs of pancreas injury (elevated lipase levels) at 2 dpi. Liu *et al*. [32] published earlier that the PKR-like endoplasmic reticulum kinase (PERK) induces degradation of the IFNAR1 chain in VSV and HCV infection, which leads to unresponsiveness to IFN-I upon virus infection. An important role of PERK action was observed in the pancreas. Mice treated with PERK inhibitor showed pancreas injury, which was due to a pathological IFNAR signaling [33].

In contrast to IFNAR-mediated protection against viral hepatitis, Bhattacharya *et al*. reported detrimental effect of IFNAR-signaling in LCMV-induced hepatitis [34]. Mice with a selective IFNAR ablation in hepatocytes were protected from LCMV-induced hepatitis, whereas in WT mice hepatitis was induced by oxidative damage. However, hepatitis in LCMV-infected WT mice was moderate and without associated hepatocyte necrosis [34], whereas in our model CVB3 infection resulted in fulminant hepatitis with hepatocyte necrosis. In mice IFNAR, and not IFNLR, signaling mediated critical ISG induction in hepatocytes, whereas in humans the situation might be different. Recently, it was shown that primary human hepatocytes expressed IFNLR and responded *in vitro* to IFN-III treatment with ISG induction that inhibited CVB3 replication [12]. We showed that CVB3 infected human hepatocytes expressed IFN-β as well as IFN-λ, and showed enhanced ISG expression. Furthermore, we detected low protein levels of IFN-β and higher protein concentrations of IFN-III in the supernatant of CVB3 infected human hepatocytes. Despite the higher concentration of IFN-III, B18R treatment of CVB3 infected hepatocytes reduced the ISG induction in CVB3 infected human hepatocytes, whereas the inhibition of the IFN-III axis had only very minor effects. Bolen *et al*. [35] compared the reactivity of similar amounts of IFN-I and IFN-III proteins on primary human hepatocytes. They found that regarding ISG induction IFN-β had the highest potency, followed by IFN-α, IFN-λ3, IFN-λ1, and IFN-λ2. This observation offers an explanation for the dominant effects of IFN-I in our experiments. Since we could only detect very minor luciferase induction in the heart of CVB3 infected IFN-β^wt/Δβ-luc^ reporter mice, it remains elusive whether local IFN-I production would suffice to induce protective effects in cardiomyocytes [20]. Hepatocyte-derived IFN-β presumably exhibits biological effects also outside the liver, e.g. in the myocard. However, the relevance of IFN-I *vs*. IFN-III in the human liver during CVB3 infection needs to be better understood before IFN-I and/or IFN-III can be used for the therapy of CVB3-induced hepatitis or myocarditis.

In conclusion, our data highlight hepatocytes as innate effector cells in CVB3 infection. Our observation that hepatic IFN-I is critical to control CVB3 infection encourages to search for individual variations in the IFN-I induction and IFNAR signaling pathways in fatal cases of neonatal hepatocyte necrosis. Furthermore, the impact of hepatic IFN-β in myocarditis and other CVB3-associated diseases has to be further analyzed.

## Materials and methods

### Mice and viruses

IFNAR1 deficient mice [36] (B6.129S2-Ifnar1^tm1(Neo)Agt^ referred to as IFNAR^-/-^) were used that were more than 10 times backcrossed to the C57BL/6JOlaHsd background. IFN-β^wt/Δβ-luc^ mice, in which the floxed IFN-β is deleted and thus the reporter is expressed ubiquitously (B6.Bruce4-Ifnb^tm2.2(luc)Lien^ [13]) and Mx2Luc BAC transgenic reporter mice [14] used for *in vivo* imaging were backcrossed to the albino (Tyr^c2J^) C57BL/6 background [37]. Conditional IFN-β^floxβ-luc/floxβ-luc^ reporter mice (B6.Bruce4-Ifnb^tm2.1(luc)Lien^ [13]) as well as IFNAR^fl/fl^ mice (B6.129SV-Ifnar^tm(flox)kal^ [38]) were intercrossed with LysM-Cre (B6.129P2-Lyz2^tm1(cre)Ifo^ [39]) and Alb-Cre mice (B6.Cg-Tg(Alb-cre)21Mgn [40]) to generate myeloid cell- and hepatocyte-specific IFN-β reporter or IFNAR-deficient mice, respectively. Mx1 mice (B6.A2G-Mx1) carried intact Mx1 alleles [41], whereas Mx1-Ifnar1-/- mice additionally lacked the type I IFN receptor (B6.A2G-Mx1-Ifnar1^-/-^, referred to as Mx1-IFNAR-/-), B6.A2G-Mx1-Il28rα-/- mice lacked the type III IFN receptor (referred to as Mx1-IFNLR-/-) and Mx1-Ifnar1-/-Il28rα-/- were double-deficient [15] (referred to as Mx1-IFNLR-/-IFNAR1-/-). For experiments, 8–14 week-old mice were used. Mice were kept under specific pathogen-free conditions on regular diet in the central mouse facility of the Helmholtz Centre for Infection Research, Braunschweig, and at TWINCORE, Centre of Experimental and Clinical Infection Research, Hannover, Germany. C57BL/6 mice were purchased from Envigo. CVB3 (strain Nancy) was generated from infectious clone p53CB3/T7 [42] and passaged twice in Green monkey kidney cells (Vero cells, ATCC CCL-81). Virus titer was determined by plaque formation on Vero cells.

### Plaque assay

Vero cells were grown to confluence in 6-well plates in MEM supplemented with 10% FCS and 1% GlutaMAX. Organs were homogenized using a FastPrep-24instrument (MP Biomedicals, Eschwege, Germany) and serial 10-fold dilutions were titrated on Vero cell monolayers. After 24 hr incubation at 37°C the cells were fixed with 5% (w/v) TCA for 2 hr at room temperature, and the monolayer was stained using 0.5% crystal violet dissolved in 5% formaldehyde, 50% ethanol, and 0.8% NaCl.

### Flow cytometry

For flow cytometry anti-CD45-Alexa Fluor 700 (BD Biosciences, Franklin Lakes, USA) was used. Data acquisition was performed using a LSR-II flow cytometer (BD Biosciences, Franklin Lakes, USA). Data were analyzed with FlowJo software (Tree Star, Ashland, USA).

### Generation of BM chimeric mice

BM cells were isolated from femur and tibia. Red blood cells were removed by RBC lysis buffer (Sigma-Aldrich, St. Louis, USA). For BM transplantation, mice were lethally irradiated with 9 Gy and received 1 x 10^7^ BM cells via tail vein injection. Mice were used 8 weeks after transplantation.

### IFN-α/β/λ determination by ELISA

Organs from perfused mice were dissected and homogenized in 20 mM Tris-HCl (pH7.3) containing 140 mM NaCl, 0.5% Triton X-100, 2 mM Na_3_VO_4_, and protease inhibitor (complete ULTRA, Sigma Aldrich, St. Louis, USA). IFN-α, IFN-β, and IFN-λ3 levels were determined from murine homogenates, serum, and supernatants of murine hepatocytes by ELISA methods following the manufacturer’s instructions (IFN-α ELISA and IFN-β ELISA: PBL Biomedical Laboratories, Piscataway, USA; IFN-λ3 ELISA: eBiosience, Thermo Scientific, Waltham, USA). IFN α, IFN-β, and IFN-λ1/2/3 levels were determined from supernatants of human hepatocytes by ELISA methods following the manufacturer’s instructions (IFN-λ1/2/3 ELISA and High sensitivity IFN-β ELISA: PBL Biomedical Laboratories, Piscataway, USA; IFN-α ELISA: eBiosience, Thermo Scientific, Waltham, USA).

### Detection of bioluminescence and *in vivo* imaging

Isoflurane anesthetized mice were intravenously injected with luciferin (100 μL of 30 mg/mL per 20 g mouse weight) and imaged on an IVIS Spectrum CT (PerkinElmer, Waltham, USA). Living Image 4.5 software was used for data analysis. For *ex vivo* detection of luciferase activity, the respective organs were homogenized in Glo Lysis Buffer (Promega, Fitchburg, USA) and bioluminescence activity was detected with a bright Glo Luciferase Assay System (Promega, Fitchburg, USA).

### Hematoxylin and eosin-staining and immunohistochemistry

Mice were perfused with PBS, organs were removed, and fixed in 4% formalin and embedded in paraffin wax. Tissues were cut at 2–3 μm thickness and sections were placed on SuperFrost Plus slides (Menzel GmbH, Thermo Scientific, Waltham, USA). For histology, a hematoxylin and eosin-staining was performed. Primary antibodies used for immunohistochemistry included rabbit polyclonal anti-mouse MX1 (1:8000, [43]), rabbit polyclonal anti-ISG15 (1:750; PB9951, Boster Biological Technology, Pleasanton, CA, USA), and monoclonal mouse anti-CVB3 mAB (clone 31A2, 1:2000, Mediagnost, Tübingen, Germany). Of note, the mouse anti-CVB3 mAb has a lower sensitivity in detecting CVB3 infection than the plaque formation assay. For antigen retrieval, samples were incubated for 30 min in the microwave with citrate buffer. For visualization of the polyclonal and monoclonal antibodies, the ABC-method (VECTASTAIN Elite ABC HRP Kit, Vector Laboratories Inc, Burlingame, USA) and the EnVision+ System-HRP (Dako/Agilent, Santa Clara, USA) was used, respectively. For the MX1 and ISG15 immunohistochemistry, a secondary goat-anti-rabbit antibody [43] was applied. CVB3 immunopositive area was quantified with AnalySIS 3.2 software (SOFT Imaging system, Münster, Germany) on a digital photomicrograph. Artefacts were manually outlined and excluded. The positive area was calculated as percentage of the outlined immunopositive area. In addition, the intensity of ISG15 staining in liver and pancreas was quantified using a semiquantitative scoring system: 0 = no staining, 1 = mild staining, 2 = moderate staining, 3 = strong staining.

### Gene expression analysis

RNA was extracted from primary human hepatocytes using NucleoSpin RNA Isolation kit (Macherey-Nagel, Germany) following the manufacturer’s instructions. RNA was reverse transcribed into cDNA using Prime-Script First Strand cDNA Synthesis Kit (TaKaRa, Kyoto, Japan) according to the manufacturer’s instructions. Primers and SYBR Green (Bioline, London, UK) were added to cDNA, and quantitative real-time PCR (qPCR) was carried out. All samples were measured as triplicates and PCR reactions were run in a LightCycler 480 (Roche, Basel, Switzerland). Target genes were normalized to the housekeeping gene hypoxanthine phosphoribosyl transferase 1 (HPRT1). The following primers were used in this study for human hepatocytes: IFNB1 (TGTGGCAATTGAATGGGAGGCTTGA; TCAATGCGGCGTCCTCCTTCTG), IFNA (CGATGGCCTCGCCCTTTGCTTTA; GGGTCTCAGGGAGATCACAGCCC), IFNL1 (AGCTTGGACCGTGGTGCTGGT; TCCAAGGCGTCCCTGGCCTTC), ISG15 (TGTCGGTGTCAGAGCTGAAG; AGAGGTTCGTCGCATTTGTC) Mx1 (ACAGGACCATCGGAATCTTG; CCCTTCTTCAGGTGGAACAC), and HPRT1 (GAACGTCTTGCTCGAGATGTG; CCAGCAGGTCAGCAAAGAATT) and murine hepatocytes: IFNb (CTGGCTTCCATCATGAACAA; CATTTCCGAATGTTCGTCCT), IFNa2 (TACTCAGCAGACCTTGAACCT; CAGTCTTGGCAGCAAGTTGAC), IFNl2/3 (AGCTGCAGGCCTTCAAAAAG; TGGGAGTGAATGTGGCTCAG), ISG15 (GAGCTAGAGCCTGCAGCAAT; TTCTGGGCAATCTGCTTCTT), and GAPDH (GTGGCAAAGTGGAGATTGTT; CTT GACTGTGCCGTTGAATT).

### Transcriptomics

For RNA sequencing (RNAseq) quality and integrity of total RNA was controlled on Agilent Technologies 2100 Bioanalyzer (Agilent Technologies; Waldbronn, Germany). The RNAseq library was generated from 500 ng total RNA using Dynabeads mRNA DIRECT Micro Purification Kit (ThermoFisher) for mRNA purification followed by ScriptSeqv2 RNA-SeqLibrary Preparation Kit (Epicentre) according to manufacture´s protocols. The libraries were sequenced on Illumina HiSeq2500 using TruSeqSBS Kit v3-HS (50 cycles, single ended run) with an average of 3 x10^7^ reads per RNA sample.

After thorough quality control, single reads were mapped to the human genome (hg19) using CLC Genomics Workbench v11.0.1 (Qiagen) with standard parameters. Normalized transcript expression is given as reads per kilobase of transcript, per million mapped reads (RPKM). A subset of 268 potential interferon regulated genes was generated by filtering transcript lists using the interferome database (www.interferome.org) search mask with filters on *in vivo | in vitro* (*in vivo*), species (homo sapiens), system (gastrointestinal tract), organ (liver), cell (hepatocytes) and a two-fold change up or down. For visualization of data GraphPad Prism version 7.04 for Windows (GraphPad Software, La Jolla California USA, www.graphpad.com) was employed.

### Isolation of primary murine and human hepatocytes

Murine hepatocytes were isolated from C57BL/6 mice using a 2-step liberase (Sigma-Aldrich) perfusion as described previously [44, 45] and cultured in Primaria 6-well plates (BD Biosciences, Franklin Lakes, USA) at a density of 1 × 10^6^ cells per well in 2 mL of HBM Basal Medium supplemented with HCM SingleQuots (Lonza, Basel, Switzerland). Hepatocytes were infected with CVB3 at MOIs 0.01, 0.1, and 1 in dublicates or triplicates and incubated for 24 hr at 37°C. Dublicates and triplicates were averaged. Human primary hepatocytes were isolated from liver specimens obtained after partial hepatectomy and cultivated as described previously (obtained from Dr. med. F.W.R. Vondran, Hannover Medical School) [46]. Human hepatocyte cultures were infected with CVB3 at MOIs of 0.1, as well as 0.3 and incubated for 24 hr at 37°C. 2 hr after infection or mock treatment hepatocytes were washed with PBS and overlayed with 500 μL fresh medium. As control uninfected cells were treated with 1 ng/mL αIFNLR (PBL Biomedical Laboratories, Piscataway, USA) or/and 100 ng/mL B18R (eBiosience, Thermo Scientific, Waltham, USA) and/or stimulated with 1 ng/mL recombinant IFN-β (PeproTech, Hamburg, Germany), 100 U/mL IFN-α (PeproTech EC, London, Great Britain), 10 ng/mL IFN-λ1 (PeproTech, Hamburg, Germany), 10 ng/mL IFN-λ2 (PeproTech, Hamburg, Germany), or 30 ng/mL IFN-λ3 (kindly provided by Rune Hartmann). Infected hepatocytes were mock treated or incubated with 1 ng/mL αIFNLR or/and 100 ng/mL B18R. Upon 12, 24, and 48 hr incubation at 37°C the supernatants and cells were harvested. Dublicates were averaged.

### Quantification of enzyme-activity

Serum alanine aminotransferase (ALT) and lipase levels were determined using commercially available kits from Fuji DRI-CHEM NX500.

### Statistical analysis

Kaplan-Meier product-limit method was used to calculate survival rates. Differences between the groups were determined using log-rank statistics. Statistical analyses were performed by One-Way ANOVA or Mann-Whitney test. A *P* value < 0.05 was considered statistically significant. For statistical analysis, GraphPad Prism Version 6.0 (Graph-Pad) was used.

### Ethics statement

#### Human subject research

Human primary hepatocytes were isolated from liver specimens obtained after partial hepatectomy of adult patients, following written informed consent of the patients (approved by the ethic commission of Hannover Medical School/Ethik-Kommission der MHH, #252–2008).

#### Animal research

All animals were handled in accordance with the German animal welfare law. Ketamin/Xylazin was used for anesthesia before organ preparation. For euthanasia mice were anesthetized with isoflurane to minimize stress and euthanized by cervical dislocation. The protocol was approved by the Niedersächsisches Landesamt für Verbraucherschutz und Lebensmittelsicherheit (Oldenburg, Germany, identification number 13/1160).

## Supporting information

S1 Fig IFN-I but not IFN-III is induced in secondary lymphatic organs upon CVB3 infection. In addition to the organ homogenates shown in Fig 1D–1F, cLN and spleen were assessed for (A) IFN-β, (B) IFN-α, and (C) IFN-λ protein levels by ELISA methods. Also serum samples were analyzed for (D) IFN-β and (E) IFN-α protein levels by ELISA methods. Pooled data from two independent experiments are shown. Bars depict median. Mann-Whitney test was used for statistical analysis, *P < 0.*05*; **P < 0.*01*.
(TIF)

S2 Fig Upon CVB3 infection IFNAR, but not IFNLR, signaling mediates ISG15 induction in hepatocytes. (A) Uninfected Mx1, Mx1-IFNAR^-/-^, Mx1-IFNLR^-/-^, and Mx1-IFNAR^-/-^IFNLR^-/-^ mice were perfused with PBS and liver and pancreas were prepared. Immunohistochemical analysis of MX1 expression within sections of liver (line 1) and pancreas (line 2) were performed. Bars = 10 μm. (B and C) Uninfected Mx1, Mx1-IFNAR^-/-^, Mx1-IFNLR^-/-^, and Mx1-IFNAR^-/-^IFNLR^-/-^ mice and mice infected i.p. with 2 × 10^4^ PFU CVB3 were perfused after 2 dpi and (B) immunohistochemical analysis of ISG15 expression within sections of liver (line 1) and pancreas (line 2) were performed. Bars = 10 μm. Representative sections of one mouse out of four ones are shown. (C) The staining intensity of ISG15 of all infected genotypes was scored using a semiquantitative scoring system. Shown are the scores for the different mice of each group and the median.
(TIF)

S3 Fig CVB3-infected mice devoid of IFNLR show similar liver pathology as WT mice. Mx1, Mx1-IFNAR^-/-^, Mx1-IFNLR^-/-^, and Mx1-IFNAR^-/-^IFNLR^-/-^ mice were infected i.p. with 2 × 10^4^ PFU CVB3 and sacrificed 2 dpi. (A) H&E staining of liver (line 1) and pancreas (line 2). Arrowheads highlight necrotic hepatocytes (coagulative necrosis). Stars depict widespread necrosis of the exocrine pancreas Bars = 50 μm. (B) Alanine aminotransferase (ALT) level were determined from serum 2 dpi (n = 4). Bars depict median. (C) Quantification of area of infected liver tissue in CVB3 immunohistochemistry sections determined by AnalySIS 3.2 software (n = 4). Bars depict median.
(TIF)

S4 Fig CVB3-infected mice with an IFNAR deletion in hepatocytes or myeloid cells show necrosis of acinar cells. C57BL/6, IFNAR^-/-^, Alb-Cre^+/-^IFNAR^fl/fl^, and LysM-Cre^+/-^IFNAR^fl/fl^ mice were CVB3 infected i.p. with 2 × 10^4^ PFU and sacrificed 0, 2, or 3 dpi (n = 3–5). (A) Pancreas sections were stained for H&E (line 1) or subjected to CVB3-specific immunohistochemistry (line 2). Necrosis of exocrine pancreas (stars) and CVB3-virus antigen (brown signal) is detected in all mice. Insert line 1: Endocrine islet cells lack morphological changes. Insert line 2: Pancreatic ducts lack CVB3-virus antigen. Bars = 50 μm. (B) Quantification of the area of infected pancreas tissue in CVB3 immunohistochemistry sections was performed by AnalySIS 3.2 software (n = 3–5). Bars depict median.
(TIF)

S5 Fig FACS analysis of primary murine hepatocytes and functional analysis of murine as well as human primary hepatocytes. (A) FACS analysis of primary murine hepatocytes from Fig 6A. In FSC-A/SSC-A analysis (left panel) hepatocytes are in the upper right gate, non-hepatic cells in the lower left gate. (B) Histogram depicts stained CD45 positive cells gated on non-hepatic cells. (C) Uninfected human hepatocytes were incubated for 24 hr with B18R and/or αIFNLR and/or stimulated with recombinant IFN-β, -α, -λ1, -λ2, or -λ3. One experiment out of two similar ones is shown. (D) Primary human hepatocytes from 4 donors were CVB3 infected at MOI 0.3, supernatants were taken after 12, 24, 48 hr and analyzed in a plaque formation assay. Representative data are shown for one donor. (E and F) Primary human hepatocytes from donor 1 and 2 (also shown in Fig 6E) were (E) CVB3 infected at MOI 0.1 for 2 hr, washed with PBS, and treated either with B18R, αFNLR, or both, (F) or were solely CVB3 infected at MOI 0.1 or MOI 0.3 for 48 hr. Cells and supernatants were harvested and analyzed either by (E) qPCR or (F) ELISA, respectively.
(TIF)
