# Supplementary figures and images for: Interferon-beta expression and type I interferon receptor signaling of hepatocytes prevent hepatic necrosis and virus dissemination in Coxsackievirus B3-infected mice
Source: PLoS Pathog. 2018 Aug 3;14(8):e1007235. doi: 10.1371/journal.ppat.1007235 (PMC6107283; doi:10.1371/journal.ppat.1007235)

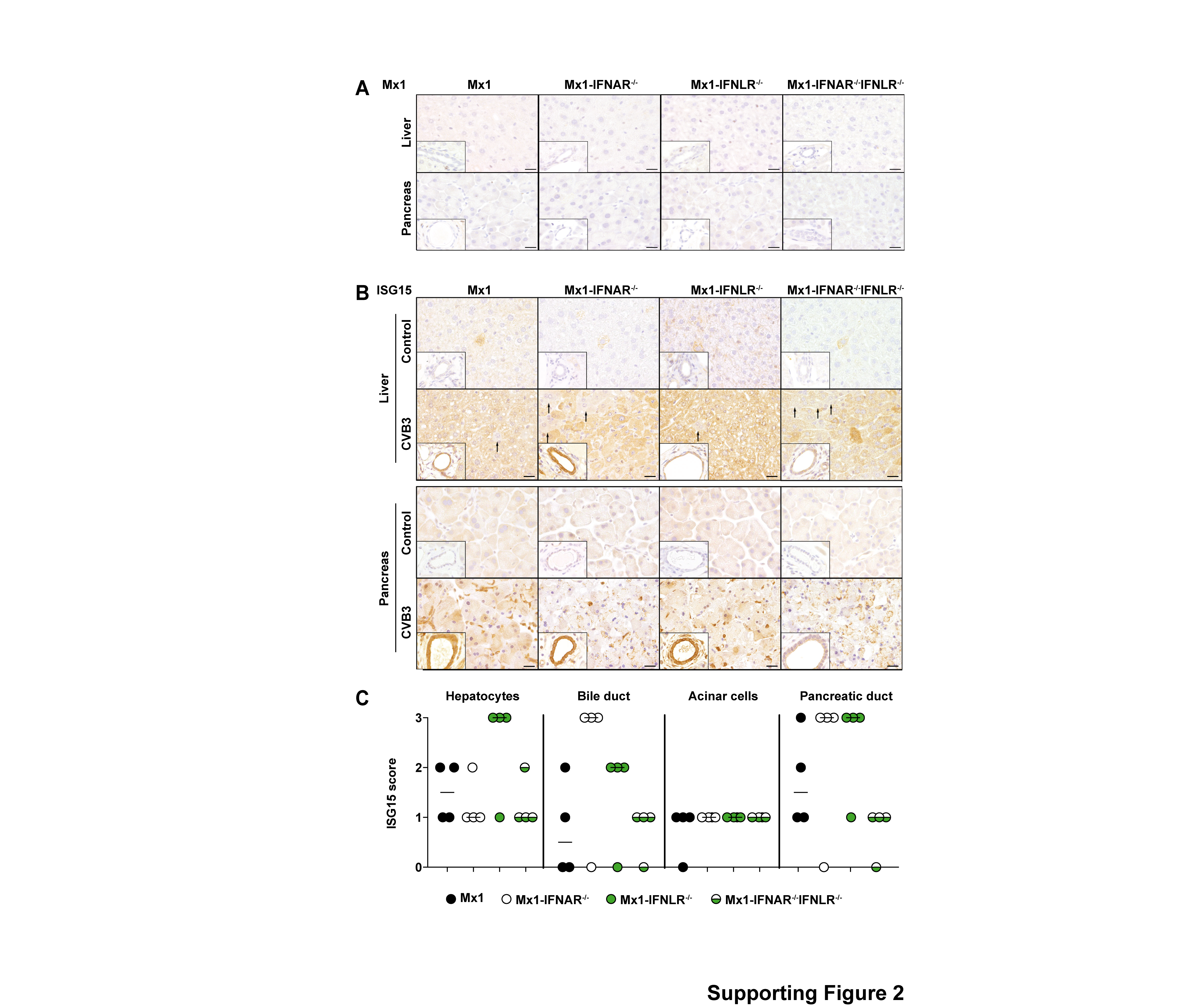

Supplement: S2 Fig — (A) Uninfected Mx1, Mx1-IFNAR-/-, Mx1-IFNLR-/-, and Mx1-IFNAR-/-IFNLR-/- mice were perfused with PBS and liver and pancreas were prepared. Immunohistochemical analysis of MX1 expression within sections of liver (line 1) and pancreas (line 2) were performed. Bars = 10 μm. (B and C) Uninfected Mx1, Mx1-IFNAR-/-, Mx1-IFNLR-/-, and Mx1-IFNAR-/-IFNLR-/- mice and mice infected i.p. with 2 × 10^4^ PFU CVB3 were perfused after 2 dpi and (B) immunohistochemical analysis of ISG15 expression within sections of liver (line 1) and pancreas (line 2) were performed. Bars = 10 μm. Representative sections of one mouse out of four ones are shown. (C) The staining intensity of ISG15 of all infected genotypes was scored using a semiquantitative scoring system. Shown are the scores for the different mice of each group and the median. (TIF) [file ppat.1007235.s002.tif]

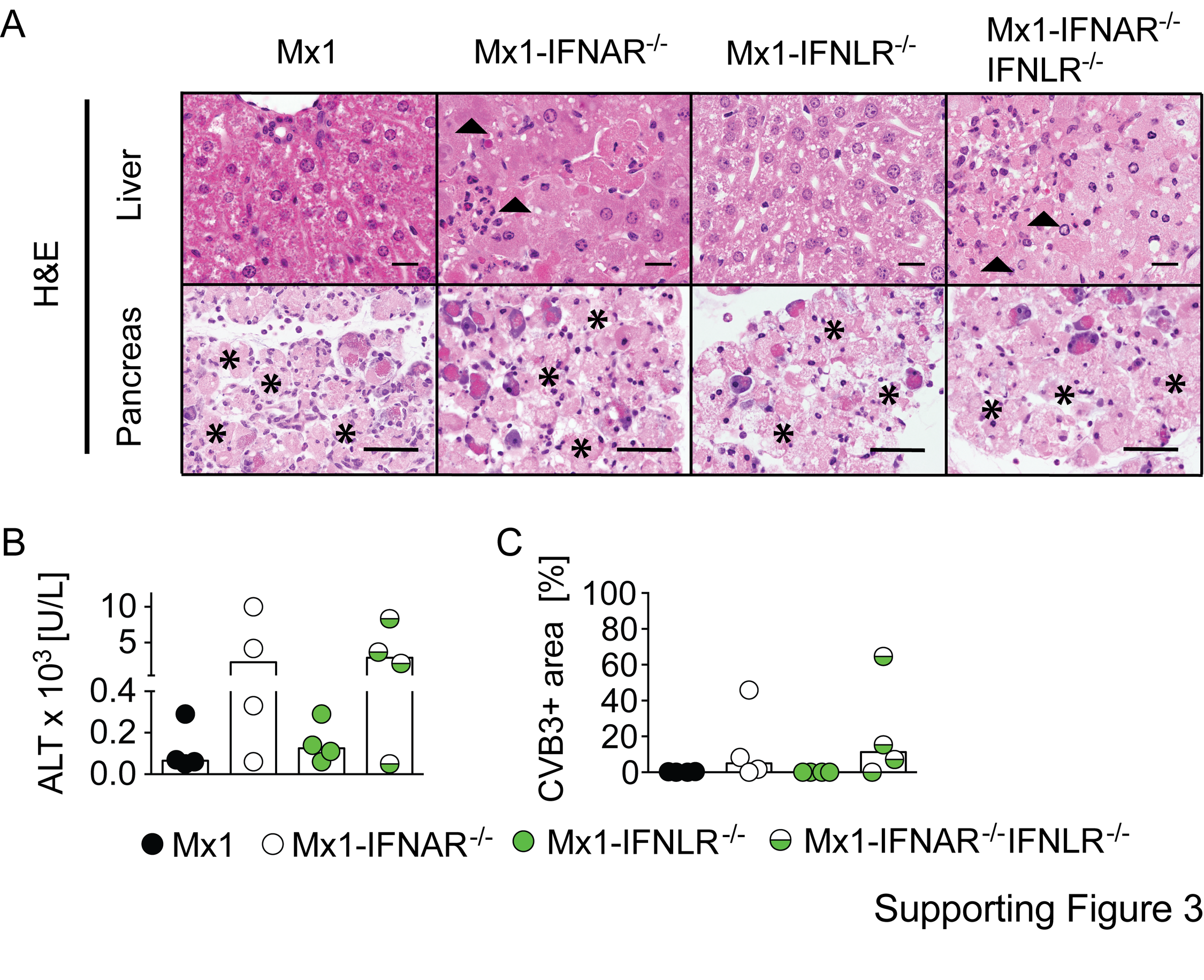

Supplement: S3 Fig — Mx1, Mx1-IFNAR-/-, Mx1-IFNLR-/-, and Mx1-IFNAR-/-IFNLR-/- mice were infected i.p. with 2 × 10^4^ PFU CVB3 and sacrificed 2 dpi. (A) H&E staining of liver (line 1) and pancreas (line 2). Arrowheads highlight necrotic hepatocytes (coagulative necrosis). Stars depict widespread necrosis of the exocrine pancreas Bars = 50 μm. (B) Alanine aminotransferase (ALT) level were determined from serum 2 dpi (n = 4). Bars depict median. (C) Quantification of area of infected liver tissue in CVB3 immunohistochemistry sections determined by AnalySIS 3.2 software (n = 4). Bars depict median. (TIF) [file ppat.1007235.s003.tif]

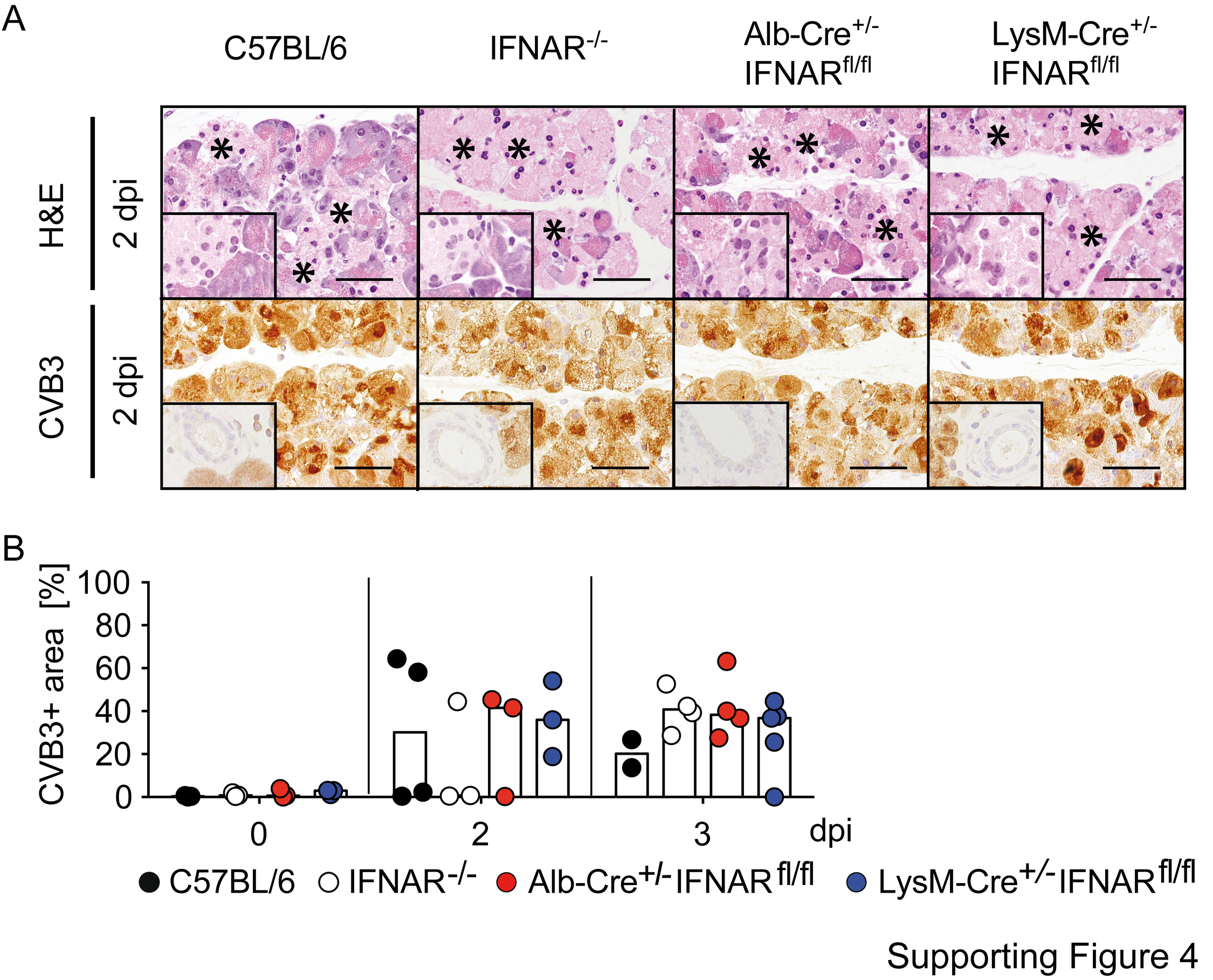

Supplement: S4 Fig — C57BL/6, IFNAR-/-, Alb-Cre+/-IFNARfl/fl, and LysM-Cre+/-IFNARfl/fl mice were CVB3 infected i.p. with 2 × 10^4^ PFU and sacrificed 0, 2, or 3 dpi (n = 3–5). (A) Pancreas sections were stained for H&E (line 1) or subjected to CVB3-specific immunohistochemistry (line 2). Necrosis of exocrine pancreas (stars) and CVB3-virus antigen (brown signal) is detected in all mice. Insert line 1: Endocrine islet cells lack morphological changes. Insert line 2: Pancreatic ducts lack CVB3-virus antigen. Bars = 50 μm. (B) Quantification of the area of infected pancreas tissue in CVB3 immunohistochemistry sections was performed by AnalySIS 3.2 software (n = 3–5). Bars depict median. (TIF) [file ppat.1007235.s004.tif]

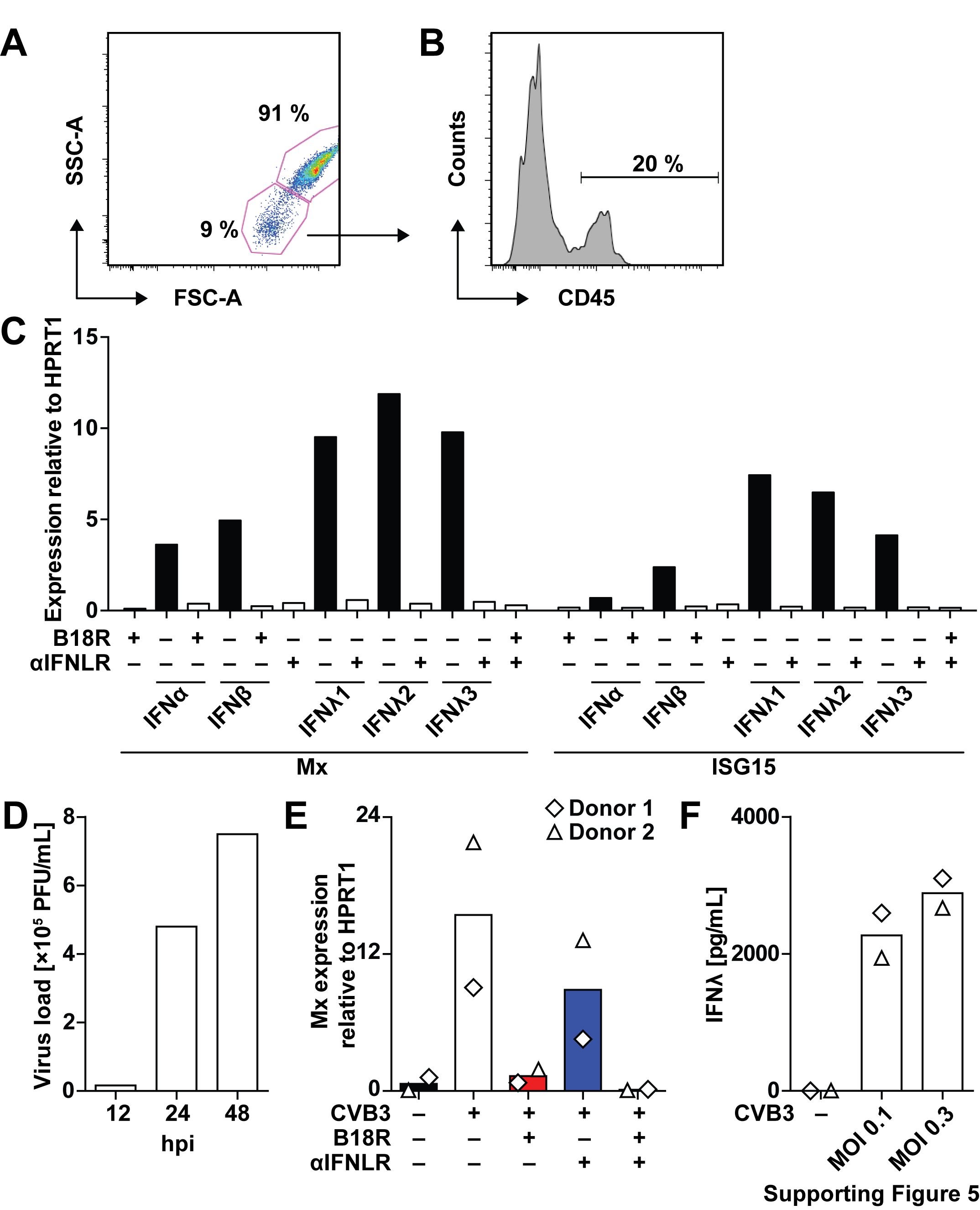

Supplement: S5 Fig — (A) FACS analysis of primary murine hepatocytes from Fig 6A. In FSC-A/SSC-A analysis (left panel) hepatocytes are in the upper right gate, non-hepatic cells in the lower left gate. (B) Histogram depicts stained CD45 positive cells gated on non-hepatic cells. (C) Uninfected human hepatocytes were incubated for 24 hr with B18R and/or αIFNLR and/or stimulated with recombinant IFN-β, -α, -λ1, -λ2, or -λ3. One experiment out of two similar ones is shown. (D) Primary human hepatocytes from 4 donors were CVB3 infected at MOI 0.3, supernatants were taken after 12, 24, 48 hr and analyzed in a plaque formation assay. Representative data are shown for one donor. (E and F) Primary human hepatocytes from donor 1 and 2 (also shown in Fig 6E) were (E) CVB3 infected at MOI 0.1 for 2 hr, washed with PBS, and treated either with B18R, αFNLR, or both, (F) or were solely CVB3 infected at MOI 0.1 or MOI 0.3 for 48 hr. Cells and supernatants were harvested and analyzed either by (E) qPCR or (F) ELISA, respectively. (TIF) [file ppat.1007235.s005.tif]
